# Atypical Fibroxanthoma

**DOI:** 10.4137/cmo.s506

**Published:** 2008-02-09

**Authors:** Akio Sakamoto

**Affiliations:** Department of Orthopaedic Surgery, Graduate School of Medical Sciences, Kyushu University, Fukuoka, Japan

**Keywords:** atypical fibroxanthoma, malignant fibrous histiocytoma, squamous cell carcinoma, malignant melanoma

## Abstract

Atypical fibroxanthoma (AFX) is a nodular dermal ulcerative lesion with a favorable prognosis. AFX most commonly occurs on sun-exposed skin in elderly individuals. AFX is characterized by its association with ultraviolet radiation, not only from a clinical aspect, but also from a molecular aspect. Making a diagnosis of AFX is challenging, and it is important to differentiate it from squamous cell carcinoma and malignant melanoma. Histological features and combined immunohistochemical markers are necessary for a definitive diagnosis (i.e., an absence of immunostaining for cytokeratins, S100 and HMB45 in AFX is helpful for excluding both squamous cell carcinoma and malignant melanoma). AFX, as well as MFH (malignant fibrous histiocytoma), is a fibrohistiocytic lesion with myofibroblastic differentiation. AFX is considered to be a different lesion from MFH. AFX and MFH might share the same pathway which determines their morphology. However, they may have different pathways in development which determine their biological behavior.

## Introduction

### History

The term, “atypical fibroxanthoma (AFX) “, was proposed by Helwig in 1963 for a lesion that was considered to demonstrate a reactive process (Helwig EB, 1963). This led to the establishment of the distinct clinicopathological features of AFX (Fretzin DF and Helwig EB, 1973).

### Clinical features

AFX has been described in two clinical settings. More commonly, the tumor occurs on sun-exposed skin in elderly individuals. The majority of tumors are on the scalp, face, ears and upper limbs (Mirza B and Weedon D, 2005). In its less common form, the tumor occurs on the limbs and trunk when there is a lack of association with sun exposure in young individuals (Dahl I, 1976). Interestingly, AFX develops in organ transplant recipients who may be in a state of immunosuppression (Hafner J et al. 1999; Kanitakis J et al. 1996; Paquet P and Pierard GE, 1996). There have been rare reports of cases of multiple tumors (Mirza B and Weedon D, 2005; Nadjem MA et al. 1988). Furthermore, it has been reported that there is a predominance in men (70% men; 30% women). The average age is reported to be 71.9 years, ranging from 29–91 years (Mirza B and Weedon D, 2005).

### Histological features

**Figure 1:**
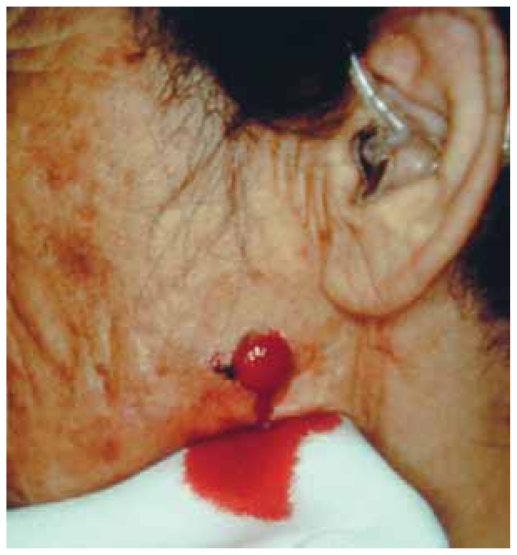
The facial lesion is protuberant and ulcerative, bleeding easily.

**Figure 2:**
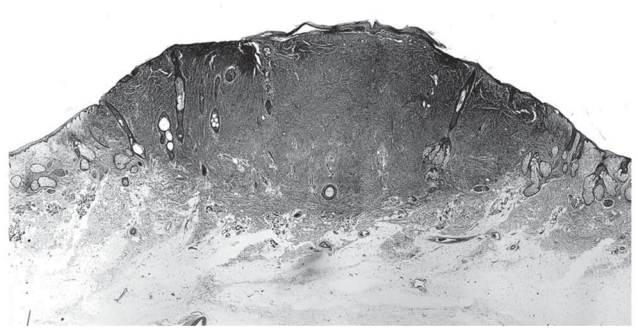
Atypical fibroxanthoma shows an exophytic cellular nodule with ulceration of the overlying epidermis. The nodule is restricted to the reticular dermis. (Hematoxylin and eosin, original magnification, x8).

AFX is a nodular dermal ulcerative lesion which typically shows rapid growth (Fig. 1). The color of AFX varies from tan to light-brown. The size is usually less than 2 cm in diameter (Enzinger FM and Weiss SW, 2001; Fretzin DF and Helwig EB, 1973) and the average preoperative size has been reported to be 1.7 cm (Seavolt M and McCall M, 2006). About half the cases are ulcerated (Mirza B and Weedon D, 2005) (Fig. 2).

**Figure 3:**
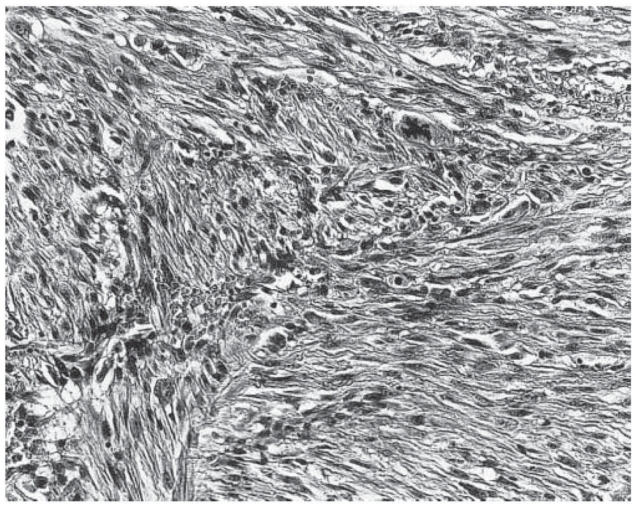
Atypical fibroxanthoma shows a proliferation of atypical spindled cells in a haphazard or disorderly pattern. (Hematoxylin and eosin, original magnification, x180).

**Figure 4:**
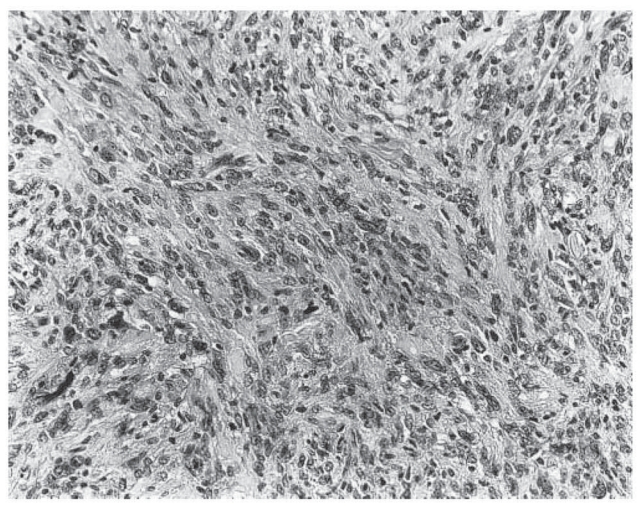
Atypical fibroxanthoma shows plump pleomorphic epithelioid cells. (Hematoxylin and eosin, original magnification, x180).

**Figure 5:**
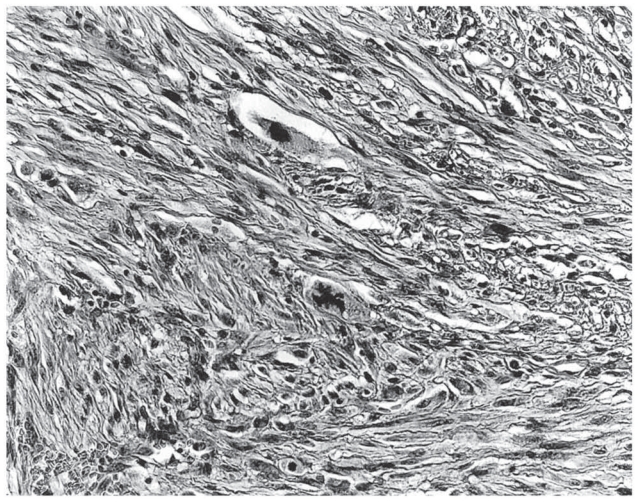
Atypical fibroxanthoma. Atypical mitosis can be observed. (Hematoxylin and eosin, original magnification, x200).

**Figure 6:**
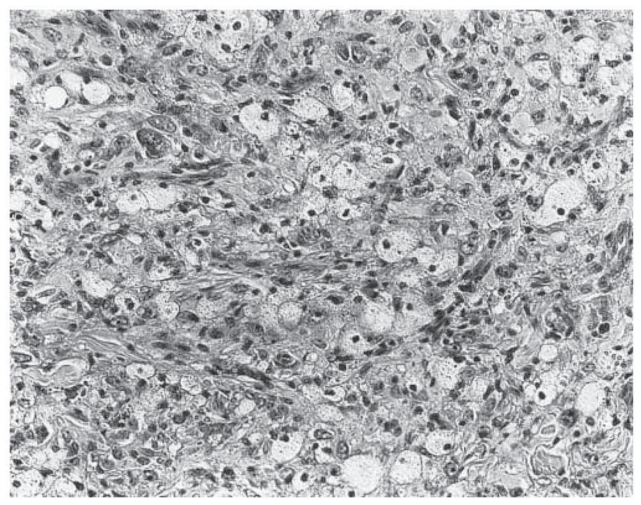
Atypical fibroxanthoma. Frequent xanthoma cells are visible. (Hematoxylin and eosin, original magnification, x200).

**Figure 7:**
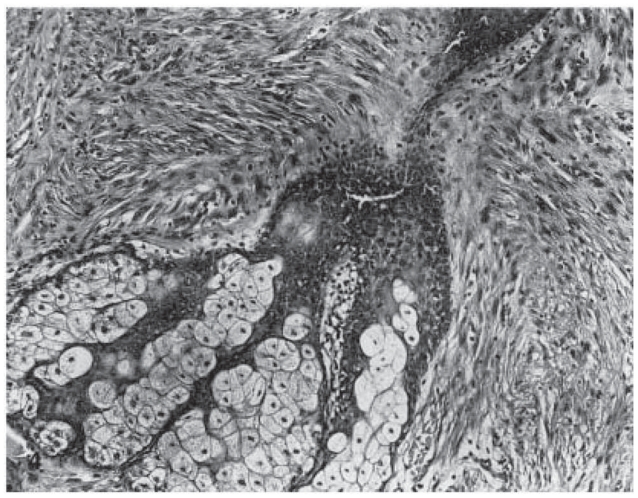
Atypical fibroxanthoma. Appendage involvement within the lesion can be observed. (Hematoxylin and eosin, original magnification, x100).

AFX is composed of spindle, plump, epithelioid and bizarre cells, in various proportions, arranged in haphazard, vaguely fascicular or storiform patterns (Figs. 3, 4). It has been reported that spindle cells predominated in 72 percent of AFX cases (Mirza B and Weedon D, 2005). Multinucleated giant tumor cells are scattered. The nuclei of the lesion may be hyperchromatic and multilobulated. In general, numerous mitotic figures, including atypical ones, can be seen (Enzinger FM and Weiss SW, 2001; Fretzin DF and Helwig EB, 1973) (Fig. 5). Some cells of the lesion may contain vacuolated and lipid-containing cytoplasm similar to xanthoma. This provided the origins for the name of atypical “xanthoma” (Dettrick A and Strutton G, 2006) (Fig. 6). Solar elastosis associated with ultraviolet (UV)-radiation has been commonly observed (Fretzin DF and Helwig EB, 1973; Sakamoto A et al. 2001a). Appendage involvement within the lesion, which is suggestive of undestroyed adnexal structures, is frequently seen (Sakamoto A et al. 2002) (Fig. 7). In addition to the classical AFX features, there have been reports of rare variants, including clear cells, osteoclastic giant cells, pigmented, osteoid, chondroid or granular cells and plaque-like cells (Crowson AN et al. 2002; Dettrick A and Strutton G, 2006; Fretzin DF and Helwig EB, 1973; Weedon D and Strutton G, 2002). Cases of AFX with prominent sclerosis and hyalinization have been also reported (Bruecks AK et al. 2003).

Since the initial description of AFX, the lesion has been a source of much controversy. Because of the histological similarity between AFX and malignant fibrous histiocytoma (MFH), AFX has been commonly regarded as a superficial variant of MFH (Enzinger FM and Weiss SW, 2001). MFH is one of the most common sarcomas occurring deep within soft tissue (Enzinger FM and Weiss SW, 2001). Most AFX cases (80%) are restricted to the reticular dermis. The lesion may extend into the upper one-third of the subcutaneous adipose tissue (Mirza B and Weedon D, 2005). Currently, AFX is considered to be different from MFH. Size and depth are important in the differentiation between AFX and MFH. If a tumor extensively involves the subcutis, penetrates the fascia and muscle, or displays necrosis or perineural and vascular invasion, it should be diagnosed as MFH (Enzinger FM and Weiss SW, 2001). Extension into deeper structures like the parotid gland has been reported (Skoulas IG et al. 2001). However, it may be better to characterize such cases as MFH (Fish FS, 1996; Sankar NM et al. 1998). Previously reported cases of AFX may need to be reviewed carefully for a more precise diagnosis based on the current clinical and histologic criteria.

When AFX is less pleomorphic, it is indistinguishable from leiomyosarcoma (Calonje et al. 1993). Histologically, being different from AFX, leiomyosarcoma has typically distinct fascicles containing cells with characteristic blunt-ended nuclei, and cytoplasmic glycogen is often abundant (Enzinger FM and Weiss SW, 2001). Dermatofibrosarcoma protuberans (DFSP) is another differential diagnosis from AFX. DFSP is a nodular cutaneous tumor and it has been considered as a fibroblastic, histiocytic and neural tumor. Microscopically, the main portion of DFSP is composed of a uniform population of slender fibroblasts arranged in a distinct, often monotonous storiform pattern. CD34 is expressed on hematopoietic stem cells and on vascular endothelial cells and it has been reported to be a marker of DFSP (Enzinger FM and Weiss SW, 2001), while the expression of CD34 is absent in AFX (Krustrup D et al. 2006).

Besides MFH and leiomyosarcoma, the major differential diagnosis includes pleomorphic and spindled variant of squamous carcinoma and malignant melanoma. Basal cell carcinoma and keratoacanthoma are also differential diagnoses. Histologically, in the differentiation between AFX and squamous cell carcinoma, there is neither junctional activity nor focal epithelial differentiation in AFX (Enzinger FM and Weiss SW, 2001). In a case of clear cell variant of AFX, additional differential diagnoses should include clear cell sarcoma (malignant melanoma of soft parts), balloon cell melanoma, metastatic renal cell carcinoma, sebaceous carcinoma and clear cell hidradenocarcinoma (Cai JP and Randall B, 2006; Crowson AN et al. 2002; Kao GF et al. 1992). Similarly, granular cell variant of AFX may need to be differentiated from granular cell tumor, including its malignant counterpart (Rudisaile SN et al. 2005).

## Differential Diagnosis in Terms of Immunohistochemistry

Making a diagnosis of AFX is challenging and the diagnosis of AFX should be made by exclusion of the differential diagnoses. Combined immunohistochemical markers are necessary for a definitive diagnosis (Table 1) (Hartel PH et al. 2006; Kanitakis J et al. 2000; Krustrup D et al. 2006). Absent immunostaining for cytokeratins, S100 and HMB45 in AFX is helpful for excluding both squamous cell carcinoma and malignant melanoma (Hultgren TL and DiMaio DJ, 2007; Mirza B and Weedon D, 2005). Cytokeratins are epithelial markers and they are positive for squamous cell carcinoma. S100 protein is widely used as a Schwann cell marker and it is also positive for malignant melanoma. HMB45 and MART-1 are melanocyte-specific markers, and these antibodies recognize gp100 and PMel17, respectively (Smith-Zagone MJ et al. 2004). However, it should be remembered that some poorly differentiated squamous cell carcinomas may not express cytokeratin, and S100 and HMB45 are not detectable in all malignant melanoma cases (Lodding P et al. 1990; Wick MR et al. 1988). Furthermore, dendritic cells within AFX may express S100 (Longacre TA et al. 1993; Ricci A Jr et al. 1988; Winkelmann RK and Peters MS, 1985). Focal HMB45 and MART-1 expression in the giant cells of an AFX has been reported (Smith-Zagone MJ et al. 2004).

CD10, or the common acute lymphoblastic leukaemia antigen (CALLA), is a cell-surface neutral endopeptidase which is expressed by lymphoid precursor cells and by B lymphoid cells of germinal center origin (Ritz J et al. 1980). CD10 is reported to be a useful marker for AFX, because of its high positivity rate (95%–100%) (Mirza B and Weedon D, 2005; Sakamoto A et al. 2002; Weedon D et al. 2005). Although one-third of malignant melanoma cases have been reported to be positive for CD10, the staining was less intense and tended to accumulate within the surrounding stroma (Hultgren TL and DiMaio DJ, 2007). On the other hand, about half the cases of squamous cell carcinoma showed positivity for CD10, but the staining was also weak in most of the cases, involving less than 25% of cells (Hultgren TL and DiMaio DJ, 2007).

CD99, or p30/32, is a glycoprotein product of the MIC2 gene. It was originally utilized in immunohistochemistry as a unique marker for Ewing sarcoma. CD99 has been reported to be a useful marker of AFX, but the positivity rate for CD99 has been reported to vary between 35% (Mirza B and Weedon D, 2005) and 73% (Monteagudo C et al. 2002) of AFX cases, although none of the squamous cell carcinoma cases have been reported to be positive for CD99 (Monteagudo C et al. 2002). However, it has been revealed that 10% (Monteagudo C et al. 2002) and 60% (Wilkerson AE et al. 2006) of malignant melanomas show positive staining for CD99.

Procollagen is secreted by fibroblasts into the extracellular matrix, where it is cleaved to form collagen. Procollagen-1 staining was strongly positive in 87% of AFX cases (Jensen K et al. 2004). On the other hand, positive staining in the tumor cells was observed in about one-third of the desmoplastic squamous cell carcinomas and desmoplastic malignant melanomas (Jensen K et al. 2004).

Taken together, these findings suggest that there is no specific sole immunohistochemical marker of AFX that would help differentiate it from squamous cell carcinoma and malignant melanoma. A combination of immunohistochemical markers is important to make a clear diagnosis of AFX.

## AFX is a Unique Entity, in Comparison to MFH

AFX, as well as MFH, is a fibrohistiocytic lesion with myofibroblastic differentiation (Sakamoto A et al. 2002). A histiocytic/macrophage marker, CD68, is positive in more than half of all AFX cases (Hultgren TL and DiMaio DJ, 2007; Smith-Zagone MJ et al. 2004). However, CD68 is also detectable in 86% of malignant melanoma cases (Pernick NL et al. 1999). As seen in MFH, AFX has immunoreactivities for several myogenic/myofibroblastic markers comprising desmin, alpha-smooth muscle actin and muscle-specific actin. Calponin and h-caldesmon are cytoskeleton-associated actin-binding proteins. They have been reported to be more specific myogenic markers. Positive or negative expression for calponin and negative expression for h-caldesmon in AFX is useful for distinguishing it from leimyosarcoma, which has smooth muscle differentiation characterized by positive findings for calponin and h-caldesmon, but such expression is shared by both AFX and MFH (Sakamoto A et al. 2002).

A high percentage of CD10 expression in AFX could be a marker from leiomyosarcoma, in which about half the cases of leiomyosarcomas are positive for CD10 (Sakamoto A et al. 2007). On the other hand, CD10 has been expressed in almost all the cases of MFH, as well as AFX (Sakamoto A et al. 2007; Weedon D et al. 2005). However, the number of AFX cases with moderate/strong or diffuse immunoreactivity for CD99 is significantly larger than that of MFH cases (Hartel PH et al. 2006). On the other hand, the LN-2 (CD74) antigen is the MHC-II complex and it is found on B cells, macrophages, monocytes, Reed-Sternberg cells and other B-cell proliferations. AFX and MFH may have been differentiated based upon positivity for CD74, in which reduced immunoexpression for CD74 in AFX is characterized compared to that in MFH (Lazova R et al. 1997). Further examination will be necessary in order to confirm the reliability of CD74 as a marker with which to differentiate between AFX and MFH (Ly H et al. 2004).

It seems that AFX and MFH share histological and immunological features. There are also similarities between the two lesions in terms of a high proliferation rate in the case of the proliferation markers of PCNA and MIB-1 (Oshiro Y et al. 1995; Sakamoto A et al. 2007), as well as similarities regarding apoptotic counts and bcl2 expression, which is a regulator protein for apoptosis (Westermann FN et al. 1997). However, from a molecular aspect, AFX is characterized by a diploid pattern, while the majority of chromosomal changes in MFH are of an aneuploid pattern (Oshiro Y et al. 1995; Worrell JT et al. 1993). H-, K-, and N-ras gene mutations are not present in AFX, whereas MFH has H- and K-ras gene mutations, although only a small number of cases was studied (Sakamoto A et al. 2001b). In a comparison between MFH and leiomyosarcoma, a previous report supported the opinion that MFH may represent a morphological pathway in tumor progression of leiomyosarcoma according to the same shared chromosome abnormality (Sabah M et al. 2005). It is also possible that AFX and MFH share the same pathway which determines their morphology, but that AFX and MFH may have different pathways of biological behavior which determine their clinical behavior. In a previous report, comparative genomic hybridization (CGH) analysis demonstrated similarities between AFX and undifferentiated pleomorphic sarcoma, or MFH. The CGH data demonstrated that genetic alterations on chromosomes 9p and 13q seem to suggest a common pathogenetic pathway (Mihic-Probst D et al. 2004).

## Molecular Mechanism

AFX typically occurs on the head and neck of sun-exposed skin. This fact has long suggested a role for sun exposure in the tumorigenesis of AFX. UV radiation from sunlight is an important risk factor for skin cancer (Aragane Y et al. 1998). DNA damage caused by UV is primarily repaired by the nucleotide excision repair (NER) pathway. The tumor suppressor protein p53 is a transcriptional activator and it is involved in cell cycle control, apoptosis and genome instability. p53 also plays a direct role in NER (Lee S et al. 1995; Reed M et al. 1995). The predominant mutation patterns of C-T transitions and CC-TT double transitions at dipyrimidine sites in the p53 gene are considered to be characterized by UV radiation (Brash DE et al. 1991; Miller JH, 1985), thereby suggesting a central role of UV radiation in the development of AFX (Dei Tos et al. 1994). The association between decreased DNA repair ability and AFX may be supported by a report showing that patients with xeroderma pigmentosum had AFX, and it is known that xeroderma pigmentosum is related to DNA repair defects (Dilek FH et al. 2000; Weedon D and Strutton G, 2002).

The formation of DNA photoproducts by UV radiation is reported to be responsible for the development of skin cancer (Pfeifer GP, 1997). Among these photoproducts, cyclobutane pyrimidine dimers (CPDs) and pyrimidine-pyrimidone (6–4) photoproducts (64PPs) are important as they are involved in mutagenesis and carcinogenesis (Hart RW et al. 1977; Moan J and Peak MJ, 1989). DNA photoproducts are removed by NER. DNA photoproducts can interfere with the binding of several important cell-cycle regulatory and DNA damage-responsive transcription factors (Tommasi S et al. 1996). It has been reported that the accumulation of DNA photoproducts may play an important role in the pathogenesis of AFX (Sakamoto A et al. 2001a).

## Prognosis and Treatment

There are no clear recommendations regarding the treatment of AFX (Seavolt M and McCall M, 2006). However, AFX is usually treated surgically, and the recurrence rate has been reported to range between 5% (Weedon D and Strutton G, 2002) and 10% (Fretzin DF and Helwig EB, 1973; Giuffrida TJ et al. 2004). Multiple local recurrence has rarely been reported (Jacobs DS et al. 1975). Metastases are uncommon and occur in approximately 1% of reported cases (Davis JL et al. 1997; Grosso M et al. 1987). However, no recurrence or metastasis was observed in 89 AFX lesions (Mirza B and Weedon D, 2005). Features associated with metastasis include recurrence, vascular invasion, large tumor, deep tissue invasion and tumor necrosis (Giuffrida TJ et al. 2004; Helwig EB and May D, 1986). It has been analyzed that these locally invasive and/or metastasizing lesions may have initially been MFH, squamous cell carcinoma or malignant melanoma, rather than AFX (Giuffrida TJ et al. 2004; Starink TH et al. 1977).

As for surgical margin, wide excision with 1 cm margins has been recommended in the past (Giuffrida TJ et al. 2004). However, there have been reports of Mohs microsurgery treatment for AFX with favorable results (Brown MD and Swanson NA, 1989; Davis JL et al. 1997; Huether MJ et al. 2001; Zalla MJ et al. 1997). Mohs microsurgery is an operation that removes the neoplasm and as little of the normal tissue as possible. During the surgery, a microscope is used to look at the neoplastic area in order to make sure that all of the neoplastic cells have been removed.

## Conclusion

AFX is now believed to be a benign lesion centered in the dermis in the sun-exposed skin of the elderly or children with xeroderma pigmentosum or other conditions with defective DNA repair. Decreased DNA repair ability might be associated as a pathogenesis in AFX, although further examinations are necessary, in order to ascertain whether this is actually true or not. Making a diagnosis of AFX is challenging. The diagnosis should be made only after applying the stringent histological criteria and a broad panel of immunostains. AFX is thought to be a different lesion from MFH. AFX and MFH might share the same pathway which determines the morphology, but they may have different pathways which determine biological activity in a tumor-specific manner.

## Figures and Tables

**Table 1:** Immunohistochemical profiles of AFX and its differentiations.

Antibody	Marker	AFX	MFH	LMS	SCC	MM	DFSP
Vimentin	Mesenchymum	+	+	+	−	+	+
Cytokeratin	Epithelium	−	−	−	+	−	−
EMA	Epithelium	−	−	−	+	−	−
S100	Schwann cell	−	−	−	−	+	−
HMB45	Melanocyte	−	−	−	−	+	−
MART-1	Melanocyte	−	−	−	−	+	−
SMA	Myocyte/myofibroblast	+/−	+/−	+	−	−	+/−
Desmin	Myocyte/myofibroblast	+/−	+/−	+	−	−	+/−
Calponin	Myocyte/myofibroblast	+/−	+/−	+	−	−	−
h-Caldesmon	Myocyte	−	−	+	−	−	−
CD10 (CALLA)	Lymphoid precursor	+	+	+/−	+/−	+/−	+/−
CD34	Endothelium	−	−	−	−	−	+
CD68	Histiocyte/macrophage	+	+	−	−	−	−
CD74 (LN-2)	MHC-II complex	+/−	+	N	N	N	+/−
CD99 (p30/32)	MIC2 gene product	+/−	+/−	−*	−	+/−	−*
Procollagen-1	Procollagen	+	+*	+/−	+/−	+/−	+

**Abbreviations:** AFX: atypical fibroxanthoma; MFH: malignant fibrous histiocytoma; LMS: leiomyosarcoma; SCC: squamous cell carcinoma; MM: malignant melanoma; DFSP: dermatofibrosarcoma protuberans; EMA: epithelial membrane antigen; SMA: smooth muscle actin; CALLA: common acute lymphoblastic leukaemia antigen; N: not reported; +: positive in most cases; +/−: positive but not always; −: negative in most cases;

* : small number of cases has been assessed.
